# To create serious movement on climate change, we must dispel the myth of indifference

**DOI:** 10.1038/s41467-022-32413-x

**Published:** 2022-08-23

**Authors:** Cynthia McPherson Frantz

**Affiliations:** Departments of Psychology & Environmental Studies, Oberlin College, Oberlin, OH USA

**Keywords:** Climate-change mitigation, Communication

## Abstract

A new study finds that Americans underestimate how many are concerned about climate change as well as support for major climate policies by nearly half, with climate policy supporters significantly outnumbering non-supporters.

## Teaser

Public underestimation of support for climate change policy is striking. Social science research tells us this is bad news, but also tells us what to do about it. Climate change communication must hammer home the overwhelming support for action.

Seventy eight percent of Americans—including 66% of Republicans—are concerned about climate change, a number that has increased dramatically in the last 3 years^1^. Climate models and recent anomalous weather events such as a “once in a millennium” heat dome in the Northwest^2^ and unprecedented wildfires in California^3^ make clear that urgent action is needed. Numerous policy^4^ and technological^5^ solutions stand waiting to be implemented, yet the United States remains in a state of fragmented and incremental progress, led primarily by NGOs, local governments, and pockets of the private sector. Why?

Gregg Sparkman and colleagues^6^ help illuminate this question by providing a crucial and thorough evaluation of public perception of climate policy support. Through an analysis of a large-N representative panel survey of US adults (from Ipsos eNation Omnibus), they demonstrate that virtually all segments of the general public dramatically underestimate the extent to which other people are concerned about climate change, as well as their support for policies to address it. Sparkman et al. explore a number of plausible (and non-mutually exclusive) explanations for this misperception, all of which warrant further study. Regardless of its cause, the magnitude of inaccuracy Sparkman et al. document is staggering: the number of Democrats who support climate policies such as a carbon tax or the Green New Deal is double the number that people estimate, and Republican support is much higher than otherwise thought.

Public misperception matters, because what we think other people think strongly impacts our own behavior^7^. Entertain this thought experiment: Imagine you are in a meeting with nine strangers, and you are quite hot. A window could be opened to let in cool air from outside. You look around the room and it seems that only 3 other people look like they might be warm. The rest seem to be quite comfortable. Would you suggest opening the window? You might, but you might not. Now imagine that instead you estimate that 6 other people look like they may be uncomfortably warm. Does that make you more likely to act? Now imagine that those 6 other people are talking about how warm it is. Suddenly it becomes obvious that you should get up and open the window.

Sparkman et al. discuss one explanation for why others influence us—namely, that social norms (what we believe others are doing and thinking) are important and powerful determinants of behavior^8^. We tend to do what we see others do. Currently, worrying about climate change is something people are largely doing in the privacy of their own minds. Based on this new data and the recent work of others on pluralistic ignorance^9^, it becomes clear that we are locked in a self-fulfilling spiral of silence. People believe that others are not concerned—or that they are even skeptical of climate change—which encourages them to refrain from discussing it with others. The lack of public discussion reinforces the norm that others are not concerned and hampers the likelihood of collective organization to address climate change. Misconceptions take on an even larger significance when we remember that those in positions of power are people too. Any misconceptions on their part influence how they behave, i.e., their willingness to support aggressive policies, make bold statements in their public outreach, or create “balanced” media coverage of climate change.

Another key determinant of human behavior with extensive empirical support is efficacy, or our belief that we can do something. If you believe the window in our thought experiment above is glued shut, you won’t even get out of your chair. Note that the window might not be glued shut! Your erroneous belief will stop you from trying anyway. Because climate change is a collective problem, taking action hinges on not just what we believe we personally can do, but on what we think others will do^7^. This is known as collective efficacy. Sparkman et al.’s data provide a siren call: Americans believe a minority are willing to support climate policies, when in fact a supermajority do. While not addressed directly by their data, the implications are clear: Creating a sense of collective efficacy, that *we* can respond effectively to climate change, is all but impossible under this level of misperception. The good news is that Sparkman et al. find that support for climate policy is, in fact, overwhelming; social norms can be changed, collective efficacy can be built, and these developments can occur quickly. It simply requires that people be exposed, over and over from sources they trust or identify with, to the fact that they are not alone in their concern and their willingness to take action^10^.

There are many scalable ways that this could happen. Individuals, faith organizations, non-profits, and businesses can make public statements via traditional means (such as letters to the editor) as well as through more modern ones (through the use of online forums such as Twitter and Instagram). Those with the opportunity to speak to wider audiences (celebrities and other public figures; elected officials; directors of parks, zoos and museums; educators) can not only speak directly to many, but can also encourage the wider public to speak up. The empowering truth is that every public statement counts, and the more diverse the voices, the more effective the message will be. Further, when every member of society has the chance to see “someone like them” speaking in favor of action on climate change, powerholders have a stronger mandate from which to work, and activists have a wellspring of concerned citizens from which to organize a movement. As Chenoweth’s^11^ research on social movements reveals, when just 3.5 percent of the population is engaged in non-violent protest, change happens. According to Chenoweth, if roughly 3 times the number of people who attended the 2017 Women’s March were well organized and persistent, “things would be totally different”^12^.

As scientists and as citizens, we need to communicate clearly and often—to regular people but especially to powerholders in politics, education, and the media—that the American people want strong and decisive action on climate change. Dispelling misconceptions about climate change policy support is a powerful lever of change. Further research such as that from Sparkman et al is critical for learning how to do this most effectively.
